# A novel and non-destructive method to examine meniscus architecture using 9.4 Tesla MRI

**DOI:** 10.1016/j.joca.2010.08.009

**Published:** 2010-11

**Authors:** M. Wang, A. Radjenovic, T.W. Stapleton, R. Venkatesh, S. Williams, E. Ingham, J. Fisher, Z. Jin

**Affiliations:** † Institute of Medical and Biological Engineering, University of Leeds, UK; ‡ School of Medicine, University of Leeds, UK; § Chapel Allerton Hospital, Leeds Teaching Hospitals NHS Trust, Leeds, UK

**Keywords:** Meniscus, MRI, Structure

## Abstract

**Objective:**

To investigate the ability of high-field (9.4 T) magnetic resonance (MR) imaging to delineate porcine knee meniscal tissue structure and meniscal tears.

**Materials and methods:**

Porcine knees were obtained from a local abattoir, and eight medial menisci with no visible defects were dissected. Lesions simulating longitudinal tears were created on two of the menisci. MR images of the menisci were obtained at 9.4 T using a three-dimensional (3D)-FLASH sequence. A detailed 3D internal architecture of the intact and injured menisci was demonstrated on high-resolution MR images.

**Results:**

High-resolution 3D MR imaging allowed visualisation of internal architecture of the meniscus and disruption to the internal structural network in damage models. The architecture of the porcine knee meniscus revealed by the MR scans appeared similar to the structures visualised by histology in previously reported studies.

**Conclusion:**

High-field MRI is a non-destructive technique to examine the internal structural components and damage/wear of meniscal tissue. It has tremendous potential in the field of functional cartilage/meniscus biomechanics and biotribology.

## Introduction

The knee meniscus, also known as ‘semi-lunar’ cartilage, is the crescent-shaped wedge of fibrocartilage present within the knee joint, situated between each femoral condyle and tibial plateau. The major components of the meniscus are water, collagen fibrils and the proteoglycan network^1–3^. It is the interaction between these components that determines the major functions of the meniscus such as load bearing and transmission, shock absorption and joint stabilisation^4–7^. Disruption of meniscal architecture as a result of degradation or trauma may affect the integrity of the extracellular matrix (ECM) organisation, shear stiffness, shear stress and resistance to tensile hoop stresses. This could result in loss of function and compromise the tribological performance. To better understand meniscal tears, it is essential to interrogate the internal structural components of the meniscus. Light microscopy and scanning electron microscopy are commonly used to investigate meniscal structure^8–11^. However, these methods require destructive preparation of samples, and can be very time-consuming if a whole-tissue 3D investigation is required.

In the field of biological and medical engineering, an understanding of the biomechanical and tribological properties of the knee meniscus and cartilage (e.g., contact stress and friction of the articulating surfaces) and their correlation with tissue degeneration and degradation such as wear and tear are important in research and development of therapies for knee diseases. To detect and characterise lesions, and to investigate tissue structural changes as a consequence of mechanical or tribological experiments, a non-invasive and high-resolution imaging technique is needed. High field high-resolution magnetic resonance imaging (MRI) may provide this solution. MRI has been used to investigate the knee meniscus for decades^12,13^, and high-field MRI scanners have been used for years to provide high-resolution images of small structure as was recently exemplified in an *in vitro* studies of cartilage wear and degradation^14^. The aims of this study were to investigate the ability of high-field (9.4 T) MRI to delineate porcine knee meniscal tissue structure and meniscal tears.

## Methods

### Sample preparation

Porcine legs were obtained from healthy large white pigs (approximately 6 months old) from a local abattoir (J.P. Penny’s, Rawdon, Leeds) within 24 h after slaughter. The average weight of the pigs was approximately 80 kg. The tibio-femoral joint was revealed after the joint capsule, ligaments, tendons and the patella were carefully removed using a scalpel. Eight medial menisci with no visible defects were carefully dissected, leaving the whole meniscal tissue with adherent capsular soft tissues. The dimensions of the eight menisci ranged from 30 to 40 mm (length), 21 to 25 mm (maximum width), and 9 to 13 mm (maximum outer rim thickness). Lesions resembling longitudinal tears were created on two of the menisci immediately after dissection using a No. 11 scalpel blade. The menisci were wrapped in phosphate buffered saline (PBS) soaked paper tissues and stored at −20°C until 6 h before MRI scans.

### MR imaging and image analysis

**Fig. 1:**
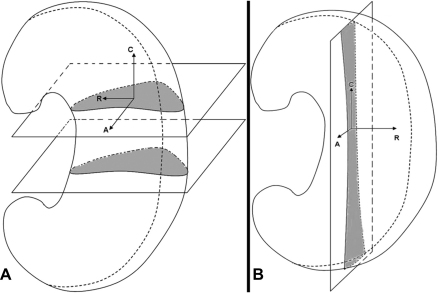
Illustration of the geometry setting of MR scans. The meniscus was put vertically in the magnet with the femoral surface towards front and the posterior horn towards the bottom. (A) Scans in the transverse orientation. (B) Scans in the sagittal orientation. (C: circumferential, R: radial, A: axial).

After thorough defrosting, PBS soaked paper tissues were removed. The meniscus specimen, with supporting pads made of polyester foam material, was put into a specimen holder/tube (diameter = 25 mm). The specimen holder was sealed with a lid. MR images of the meniscus were obtained at 9.4 T (Bruker AVANCE™ II 400 MHz laboratory NMR system) at temperature of 22 ± 0.6°C. The samples were scanned using a 3D-FLASH sequence (3D Fast Low Angle Shot; TR/TE/FA, 38/3.3/15°; scan duration ranged from 10 h 32 min to 33 h 12 min). The geometry setting of the scans is shown in Fig. 1. The in-plane field of view (FOV) ranged from 22 × 22 to 33 × 33 mm; matrix size (*Xx*, *Yx*, *Z*) was 512, 512, 64 to 256 voxels; slab thickness ranged from 5.4 to 33 mm; in-plane spatial resolution ranged from 43 to 64 μm; in the slice direction the spatial resolution ranged from 64 to 312 μm; and number of averages ranged from 10 to 18.

The signal-to-noise ratio (SNR) of MR images was measured using the Analyze™ software. A square region (size 20 voxel × 20 voxel) of the typical intrameniscal high signal area was selected, and the mean value of the signal intensity was calculated. A largest possible region outside the meniscus in the image background was also selected, and the standard deviation of the background noise intensity was calculated. The SNR was then calculated by dividing the mean signal intensity by the standard deviation of the background noise intensity. For each sample, three different images were randomly chosen for SNR measurement, and the mean value of SNR and standard deviation was calculated.

## Results

**Fig. 2:**
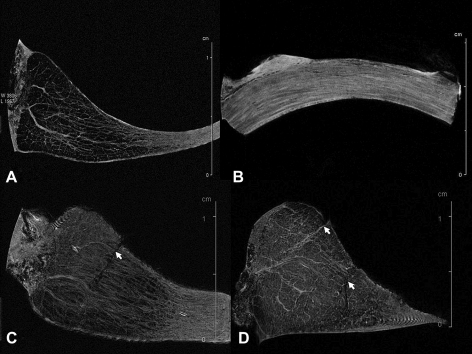
Two-dimensional MR images showed the internal structure of porcine knee menisci. (A) Transverse MR plane showed the radially arranged structure. Spatial resolution, 43 × 43 × 84 μm. (B) Sagittal MR plane showed the circumferentially arranged structure. Spatial resolution, 59 × 59 × 312 μm. (C, D) Transverse MR planes showed tears and disruption of networks of the meniscal tissue (arrows). Spatial resolution, Image C: 43 × 43 × 125 μm; Image D: 47 × 47 × 86 μm.

The 3D-FLASH 9 T MRI allowed delineation of intrameniscal structures (Fig. 2). A 16-h FLASH 3D scan achieved a fine in-plane spatial resolution (43 × 43 μm) with the mean SNR value of 16.4 with a standard deviation of 1.34. The images from a 33-h scan have a spatial resolution of 47 × 47 × 86 μm with the mean SNR of 40.58 with a standard deviation of 1.74. The intrameniscal structure could be clearly observed on MR images. A fine network of high signal lines within the meniscal tissue (Fig. 2) showed an appearance reminiscent of the radially oriented tie fibres and the circumferentially oriented fibres that have been observed using microscopy techniques^8,9,11^. High-resolution MR images also allowed visualisation of the simulated meniscal tears, particularly the disruption to tissue network [Fig. 2(C–D)].

## Discussion

In the field of functional meniscus biomechanics and biotribology, it is of value to be able to investigate the natural tissue matrix of the specimen after tribological experiments, in order to assess the effects of loads and motions in fundamental experiments. This may involve laboratory experiments on human or animal knee joints mimicking walking or running to help understanding of the role of knee meniscus in the functioning of the knee joints and it requires a non-destructive method which enables visualisation of the specimen in its native form in three dimensions. The method should also be quick enough to prevent the specimen dehydrating. Histology requires dissection and sectioning of the meniscus, it is destructive and time-consuming. High-field 3D MRI allowed preservation of the integrity of tissue and provided a virtual cross-sectional view of the internal architecture of the entire meniscus at any desired angle. The native meniscus was subjected to an MRI scan immediately following dissection. A half-day scan provided spatial resolution and SNR ratio that were sufficiently high to demonstrate 3D meniscal architecture. Furthermore, the application of accelerated MRI acquisition techniques may reduce the required scanning time, whilst preserving image quality.

However, there are several limitations of this study. Firstly, the image finding could not be deciphered as clearly as histology, though the structures seen on the MR images showed a strong resemblance to the actual fibrous structures revealed by microscopy in other reports. The porcine knees used in this study were from skeletally immature 5- to 6-month-old animals. It is known that immature menisci were more vascularised than mature menisci. Therefore, it would be highly possible that part of the intrameniscal signals were from blood vessels, which were difficult to be differentiated from fibrous structures on the images. Secondly, dehydration and deformation of the tissue could occur during a long scan, which would result in blurring of the images. In addition, the meniscal damage created in this study had limitations to mimic the naturally occurred tears, hence the disruption of the meniscal architecture we observed might differ from the natural tear signals.

In conclusion, high-field MRI is a promising technique to non-destructively examine the internal structural components and damage/wear of meniscal tissue in 3D. It has tremendous potential in the field of functional cartilage/meniscus biomechanics and biotribology. Future study will involve characterisation of meniscal components by contrast enhanced imaging and MRI–histological correlation study, characterisation of wear volume, and minimisation of the effect of dehydration and deformation using more sophisticated sample preparation and faster imaging protocols.

## Author contributions

All authors have made substantial contributions to the conception and design of the study, interpretation of the image finding, critical revising of the draft, and final approval of the version to be submitted. Manyi Wang (manyiwang@hotmail.com) takes responsibility for the integrity of the work as a whole, from inception to finished article.

## Conflict of interest

None of the authors of the above manuscript has declared any conflict of interest within the last 3 years which may arise from being named as an author on the manuscript.
